# Supplementary material for: Pleckstrin homology domain-containing protein PHLDB3 supports cancer growth via a negative feedback loop involving p53
Source: Nat Commun. 2016 Dec 23;7:13755. doi: 10.1038/ncomms13755 (PMC5196188; doi:10.1038/ncomms13755)
Supplement: Supplementary Information — Supplementary Figures 1-7 [file ncomms13755-s1.pdf]

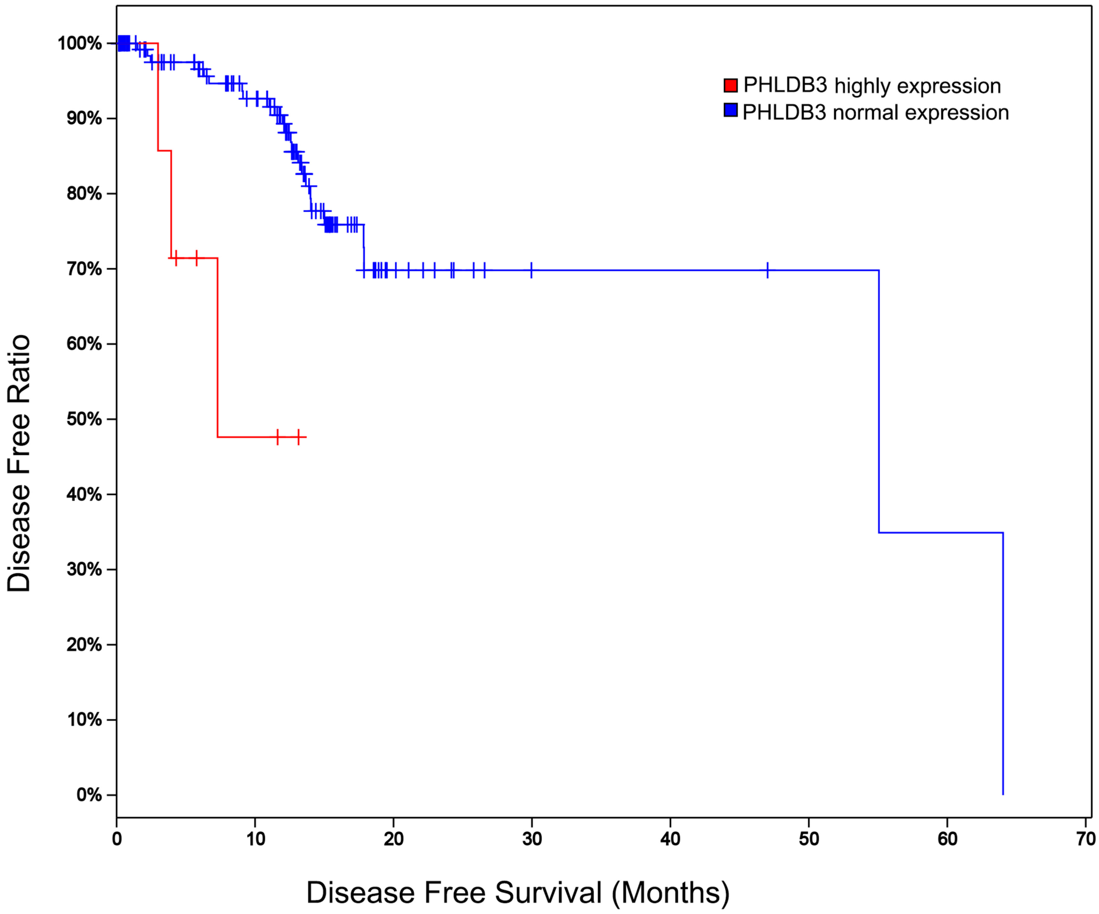

Supplementary Figure 1. High expression of PHLDB3 is associated with PFS of Stomach Adenocarcinoma patients. The PHLDB3 expression and DFS survival data of Stomach Adenocarcinoma study (TCGA, Nature 2014) were downloaded from the cBioPortal for Cancer Genomics (http://www.cbioportal.org/). Logrank Test, P-Value: 8.911e-4.

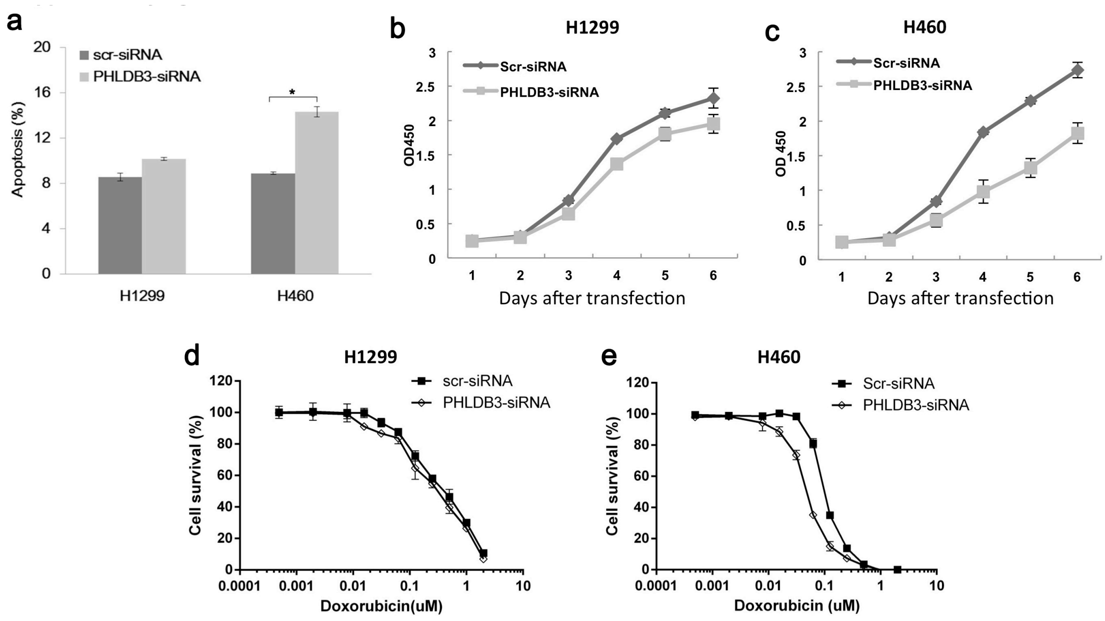

Supplementary Figure 2. PHLDB3 knockdown induces apoptosis and inhibits cell proliferation of lung cancer cells, and sensitizes these cells to chemotherapy. **(a)** The effect of PHLDB3 knockdown on apoptosis of H1299 and H460 cells. H1299 and H460 cells cells were transfected with PHLDB3 or scramble siRNA and harvested 72h post-transfection for flow cytometry analysis. Quantification of Sub-G1 population is shown. Data represent mean ± s.e.m. of triplicate experiments.*P<0.01 by two-tailed t-test. **(b) and (c)** The effect of PHLDB3 knockdown on cell growth. H1299 and H460 cells were transfected with PHLDB3 or scramble siRNA and cell viability was evaluated every 24 h by CCK8. (d**) and (e)** Knockdown of PHLDB3 sensitizes lung cancer cells to chemotherapy. H1299 and H460 cells were transfected with PHLDB3 or control siRNA, and seeded in 96-well plates next day. Doxorubicin was supplemented for 72h before cell viability detection by CCK-8.

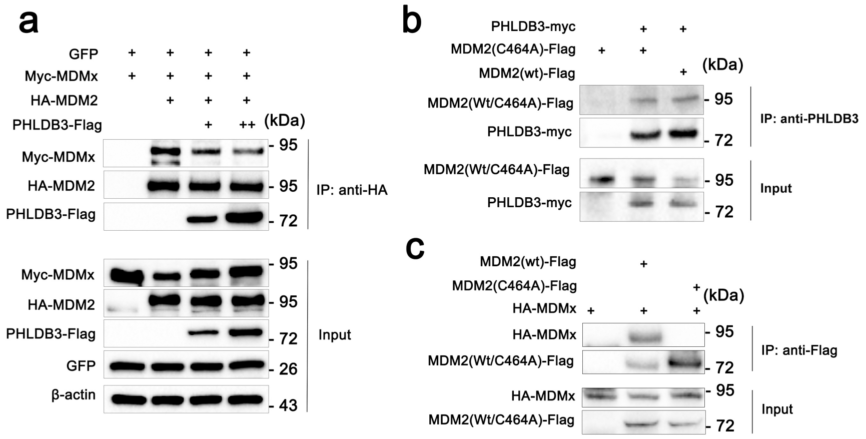

Supplementary Figure 3. PHLDB3 competes with MDMX for binding to wild type, but not C464A mutant, MDM2.

(**a**) Ectopic PHLDB3 reduces the interaction between MDM2 and MDMX in a dose dependent manner. HEK293 cells were transfected with plasmids encoding MDMX and/or MDM2 and/or different amounts of PHLDB3 plasmid as indicated followed by Co-immunoprecipitation assays using antibodies as indicated. (**b**) The interaction between ectopic PHLDB3 and wild type or mutant (C464A) MDM2. HEK293 cells were transfected with plasmids encoding PHLDB3 and/or wild type or C464A mutant MDM2 as indicated followed by Co-immunoprecipitation assays using antibodies as indicated. (**c**) MDMX interacts with wild type, but not C464A mutant, MDM2. HEK293 cells were transfected with plasmids encoding MDMX and/or MDM2 or MDM2 (C464A) as indicated followed by Co-immunoprecipitation assays using antibodies as indicated.

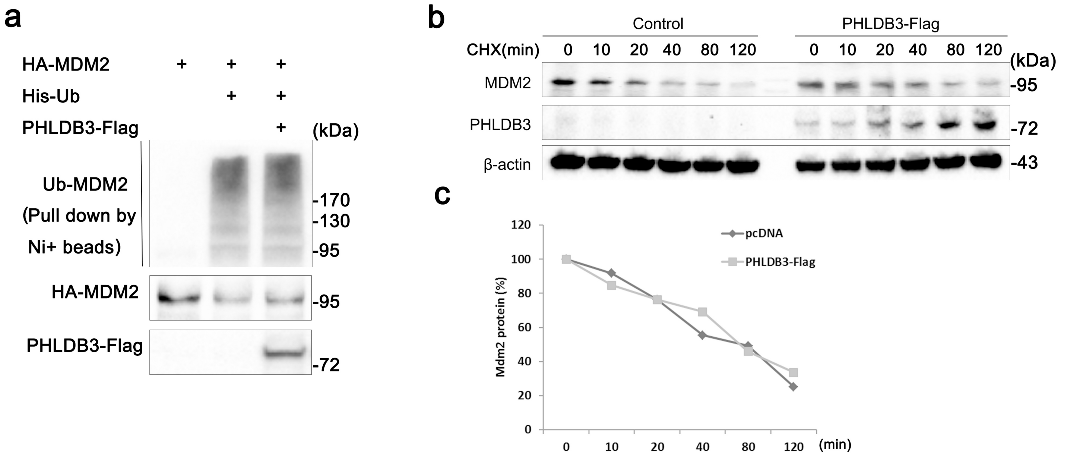

Supplementary Figure 4. PHLDB3 does not affect MDM2 ubiquitination and stability.

(a) Ectopic PHLDB3 does not appear to affect the ubiquitination of MDM2. HCT116$^{p53+/+}$ cells were transfected with combinations of plasmids encoding EGFP, HA-MDM2 or PHLDB3-Flag followed by immunoblotting using antibodies as indicated. MG132 was supplemented to the medium for 6 h. **(b)** and **(c)** PHLDB3 overexpression does not appear to affect MDM2's half-life. HCT116$^{p53+/+}$ cells transfected with PHLDB3 or pcDNA3 vector for 36h were treated with 100 µg/ml of CHX and harvested at different time points as indicated. The MDM2 protein level was detected by immunoblotting (**b**), and quantified by densitometry and plotted against time to determine MDM2's half-lives (**c**).

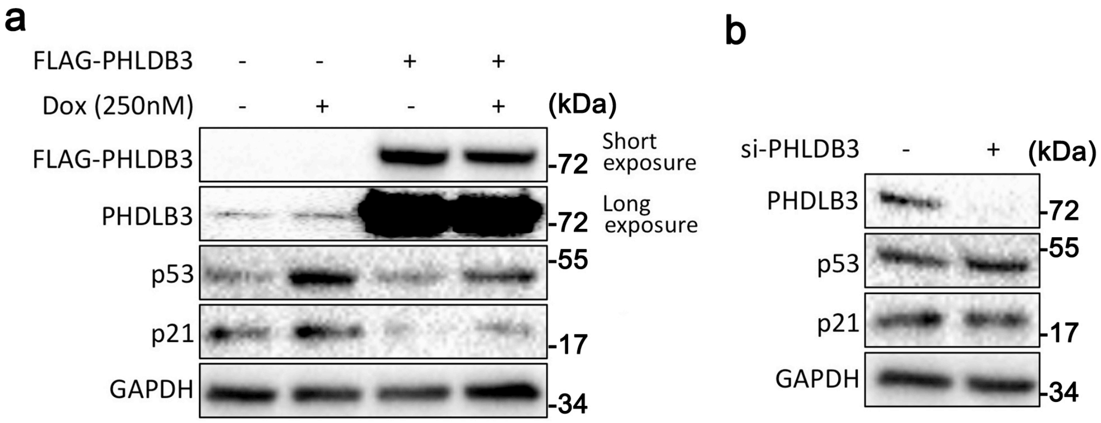

Supplementary Figure 5. Effect of PHLDB3 on p53 pathway in WI-38 cells.

(**a**) WI-38 cells were transfected with vector DNA or FLAG-PHLDB3, and 24 h after transfection the cells were treated with vehicle or 250 nM doxorubicin (Dox) for 18 h and then subjected to immunoblotting analysis. (**b**) WI-38 cells were transfected with scramble or PHLDB3 siRNAs and subjected to immunoblotting analysis 72 h after transfection.

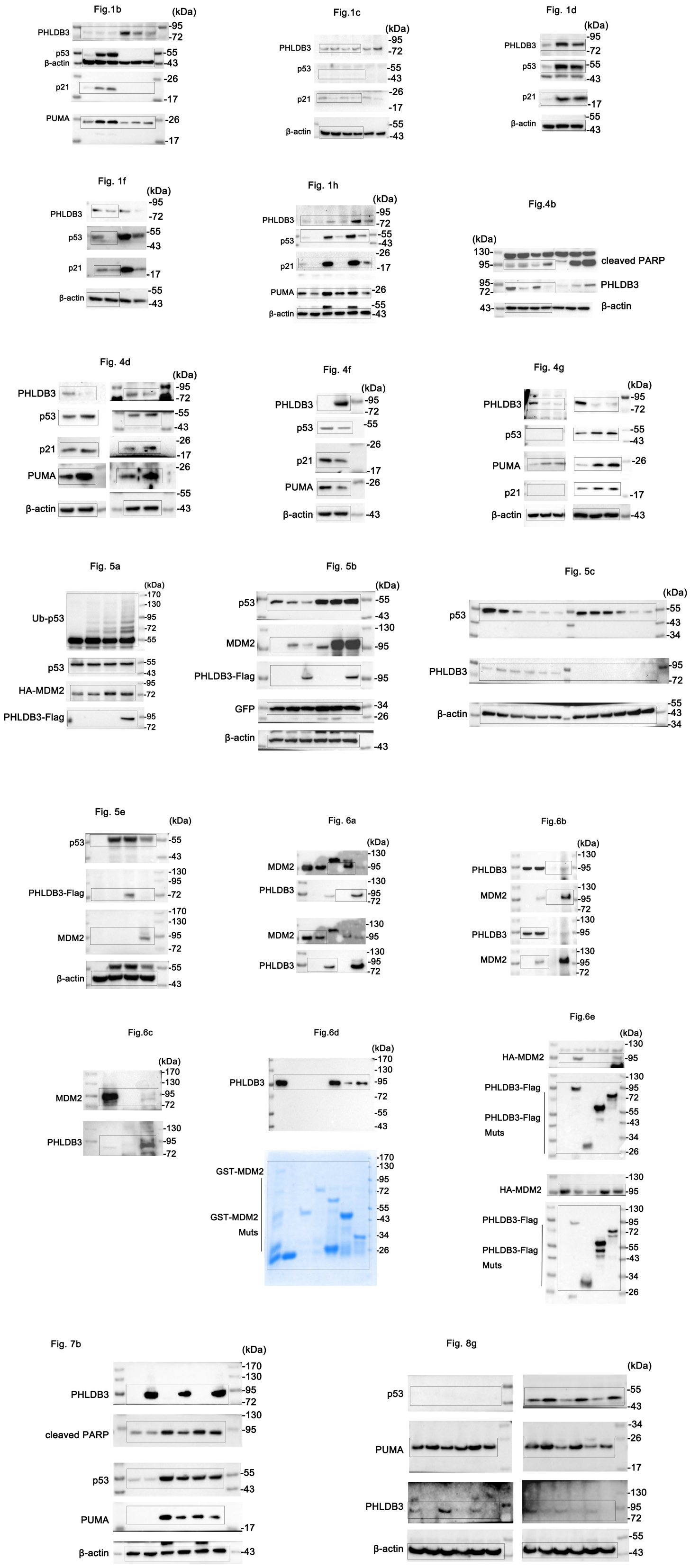

Supplementary Figure 6. Original scans of Western blotting corresponding to the main figures.

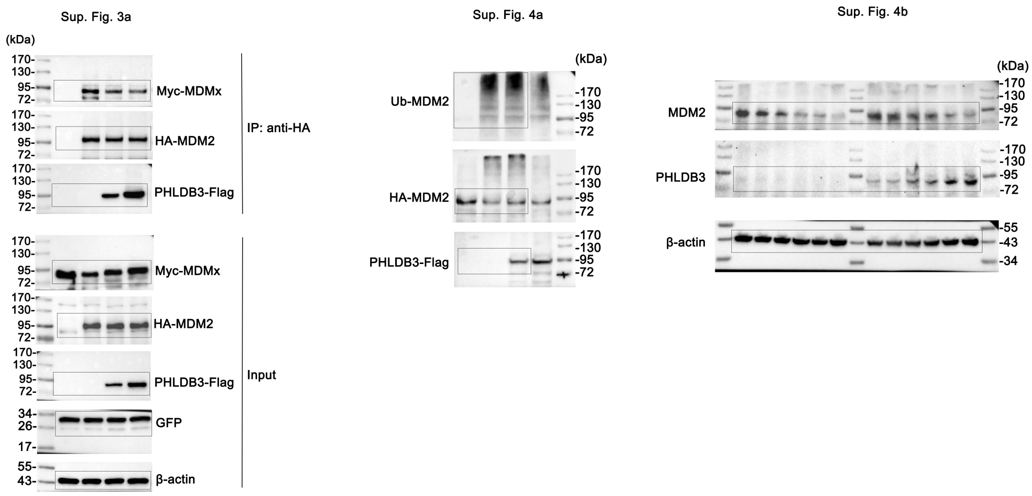

Supplementary Figure 7. Original scans of Western blotting corresponding to supplementary figures 3 and 4.
